# Recurrent Hydropneumothorax After COVID-19

**DOI:** 10.7759/cureus.36208

**Published:** 2023-03-15

**Authors:** Vashistha M Patel, Shreya V Patel, Kyle Singletary, Lauren Pacheco

**Affiliations:** 1 Internal Medicine, Brookwood Baptist Health, Birmingham, USA

**Keywords:** exploratory thoracotomy, voriconazole, covid-19, recurrent hydropneumothorax, pleuropulmonary aspergillosis

## Abstract

A 60-year-old male with a past medical history of heart failure with reduced ejection fraction, obstructive sleep apnea, atrial flutter, and hypertension initially presented to the emergency department with a chief complaint of shortness of breath. He was diagnosed with COVID-19-induced acute hypoxic respiratory failure. Before his presentation to the emergency department, he was treated with a brief course of hydroxychloroquine, azithromycin, and prednisone. His initial hospitalization was relatively uncomplicated. He then presented back to the emergency department approximately five months later with chief complaints of continued dyspnea and increased work of breathing. On this presentation, he was noted to have a right-sided pneumothorax with a moderate right-sided pleural effusion. The effusion was drained through CT (computed tomography)-guided catheter insertion. Pleural fluid culture and sensitivity were negative, and a cartridge-based nucleic acid amplification test (CBNAAT) was not performed. He was discharged a few days later to home. Over the next several weeks, the patient had recurrent admissions and chest tube placements for unresolving hydropneumothorax. He eventually had a right-sided posterolateral thoracotomy performed. The tissue sample from the thoracotomy was noted to have positive gram staining for fungal hyphae consistent with aspergillosis. This was initially considered a contaminant and not treated with antifungal medication. Unfortunately, after the thoracotomy, the patient continued to have complications including subcutaneous emphysema and recurring hydropneumothoraces. He was taken for another procedure after a repeat CT showed intercostal herniation of the pleura between the fifth and sixth ribs. The herniation was excised, and the pleura was repaired. This pleural tissue was then sent to pathology and noted to have non-caseating granulomas consistent with aspergillosis. At this time, the patient was started on voriconazole. After initiating this medication, the patient's last chest x-ray showed stable findings of his chronic disease process with no new or worsening hydropneumothorax.

## Introduction

As already well documented in the medical literature, immunocompromised hosts are at an increased risk for developing fungal infections. Pulmonary infection from Aspergillus species can manifest as a wide spectrum of illnesses depending on the immune status of the host [1,2]. The important and different clinical forms of these are allergic bronchopulmonary aspergillosis, chronic necrotizing Aspergillus pneumonia, aspergilloma, and invasive aspergillosis [3]. The classic triad of fever, pleuritic chest pain, and hemoptysis with imaging showing cavitary nodules or abscesses have been described as the presenting symptomatology of pulmonary aspergillosis. However, more case reports and newer data indicate increased incidences of presentations that are atypical to this, particularly in neutropenic patients [2]. These patients were noted to have pulmonary imaging more consistent with acute respiratory distress syndrome, bacterial pneumonia, and pleural effusions. We present to you a case of pleural aspergillosis following COVID-19-induced viral pneumonia.

## Case presentation

A 60-year-old male with a past medical history of heart failure with reduced ejection fraction, obstructive sleep apnea, atrial flutter, and hypertension initially presented to the emergency department (ED) with the chief complaint of shortness of breath. He was diagnosed with COVID-19-induced acute hypoxic respiratory failure. In the ED, he had hypoxia at 87% with other vitals within normal limits. He was treated with a brief course of hydroxychloroquine, azithromycin, and prednisone for five days as an outpatient. His hospitalization was relatively uncomplicated, and he was discharged a few days later to home on 2 L of oxygen. The patient had a follow-up appointment approximately five months later with the pulmonology outpatient clinic and reported a salty taste in his mouth since contracting COVID-19 infection. He was using an oxygen concentrator at home without any relief from dyspnea. Outpatient treatment with inhaled albuterol with fluticasone and vilanterol did not result in symptom relief. Inhalers were changed to budesonide with glycopyrrolate and formoterol with no resulting benefit as well. He was compliant with a continuous positive airway pressure machine with 2 L of supplemental oxygen while asleep. He denied using tobacco in any form, but his occupation was related to welding work and might have had some exposure in the past. The patient had lived with a heavy smoker for 11 years over 20 years ago. He was treated for a possible sporotrichosis with ketoconazole for 10 days by his primary care provider a few months ago. Chest X-ray performed in the clinic showed a small right-sided pneumothorax not requiring hospitalization and was treated conservatively due to stable condition.

**Figure 1:**
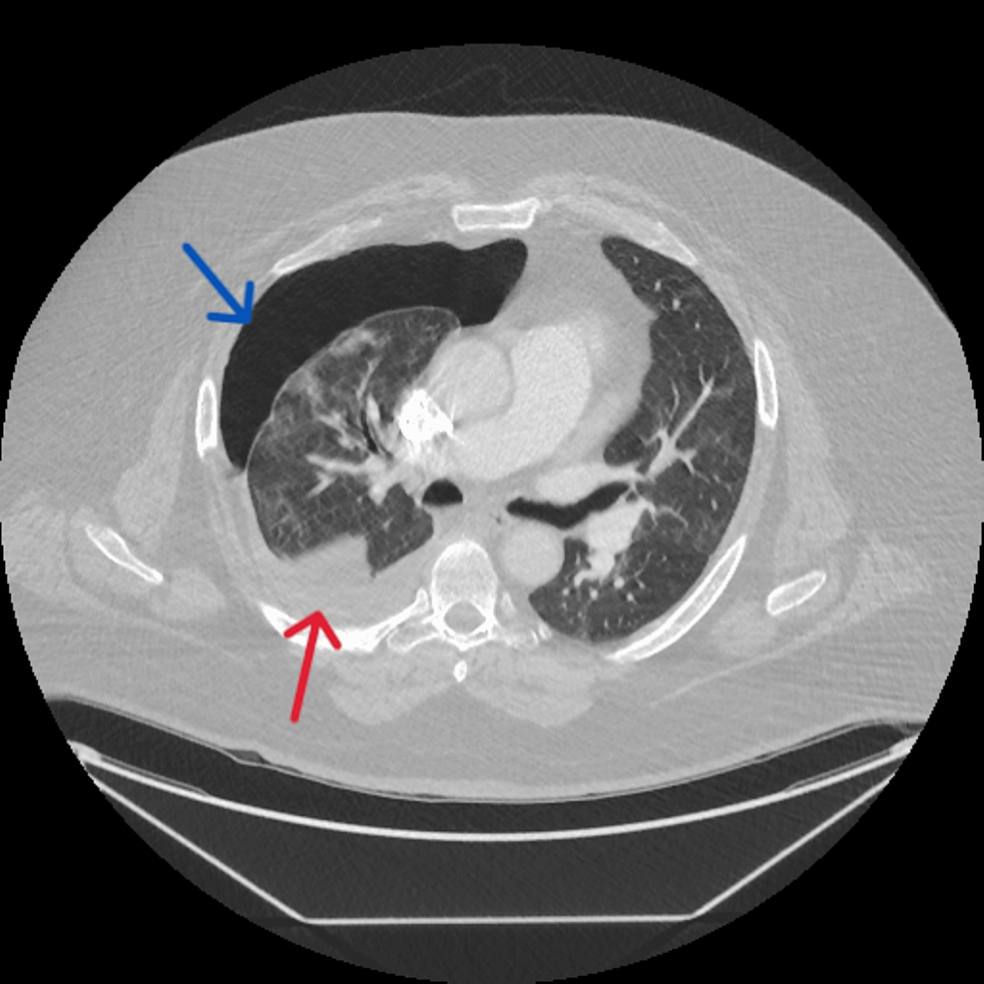
Computed tomography angiogram (CTA) chest The blue arrow shows that 40-50% right-sided pneumothorax has developed without obvious leftward cardio mediastinal shift. The red arrow shows moderate-sized layering right pleural effusion.

Following a week, he continued to have worsening dyspnea and increased work of breathing, which was evaluated by a computed tomography angiogram (CTA) chest and was noted to have a large right-sided 40-50% pneumothorax with a moderate right-sided pleural effusion in ED (Figure 1).

On this presentation, he was afebrile with mild hypotension at 104/65 mm Hg, tachycardic at 110 beats per minute, and tachypneic at 24 breaths per minute with 94% oxygen saturation on room air. He was alert and oriented with no acute distress and was able to phonate in complete sentences. Respiratory examination revealed decreased breath sounds on the right upper lung field compared to the left. No wheezing was appreciated, and on the first surgical intervention, a right-sided small-bore chest tube catheter was inserted by the surgical team. Cardiovascular examination revealed tachycardia and irregularly irregular rhythm; no rub or gallop was heard, and equal pulses in all extremities, normal peripheral perfusion, and minimal edema in bilateral lower extremities were noted. Initial Laboratory findings were remarkable for leukocytosis at 12,700/microliter with a neutrophilic predominance and normal hemoglobin, hematocrit, and platelets. Electrolytes were within normal limits. His COVID-19 polymerase chain reaction (PCR) screen was negative. Pneumocath was placed in the right pleural cavity in the ED with subsequent improved right pneumothorax to 10-15% on repeat chest X-ray. On day 2, CT-guided placement of a locking pigtail catheter into the right pleural space was performed to drain the pleural effusion and was removed on day 5 without complications. Pleural fluid studies were negative. He was discharged with a close follow-up with the pulmonology clinic. The patient did not receive any systemic antibiotics and remained hemodynamically stable with no signs of infection and was continued on systemic anticoagulation for the history of atrial flutter. He was discharged to home on 2 L of supplemental oxygen on day 5.

He presented back to the ED within one week for worsening dyspnea on minimal ambulation. He denied any fever, chills, or cough. Laboratory studies were significant for elevated pro-brain natriuretic peptide at 1530, negative troponin, and negative COVID-19 PCR. Complete blood count and electrolytes were unremarkable. Vital signs were only significant for mild tachypnea at 23 and a physical examination with decreased breath sounds in the right base, no wheezing, and symmetric expansion was noted. Chest X-ray showed 5-10% right apical pneumothorax with increasing right pleural fluid representing hydropneumothorax with worsening of right basilar fluid and opacities increased since the prior exam. Cardiothoracic surgery was consulted for surgical evaluation with plans for video-assisted thoracoscopic surgery. Approximately 1500 mL of serous effusion was evacuated from the pleural space. Initially, with the thoracoscope insertion, no lung was identified as it was encased in a fibrous rind. Thoracotomy was then undertaken with pan-lobar decortication. Marked emphysematous changes to the lung and an old small abscess in the right posterior basilar segment were seen along with well-organized rind over all lobes. Due to the intense reaction, the usual multiple rents were created. After removing the rind, the lung was reinflated and expanded nicely. The chest cavity was copiously irrigated and closed. 

The surgical pathology report showed acute and chronically inflamed fibrovascular connective tissue with necrosis and patchy fibrinopurulent exudate of the right pleura. The tissue sample from the thoracotomy was noted to have positive Gram staining for fungal hyphae consistent with aspergillosis. This was initially considered a contaminant as there were no clinical signs of underlying infection and, thus, not treated with antifungal medication. Following the patient’s improvement in the overall condition with surgery and diuresis, the drain was removed on day 6 of admission and the patient was discharged. Pleural fluid studies were negative for any culture growth.

**Figure 2:**
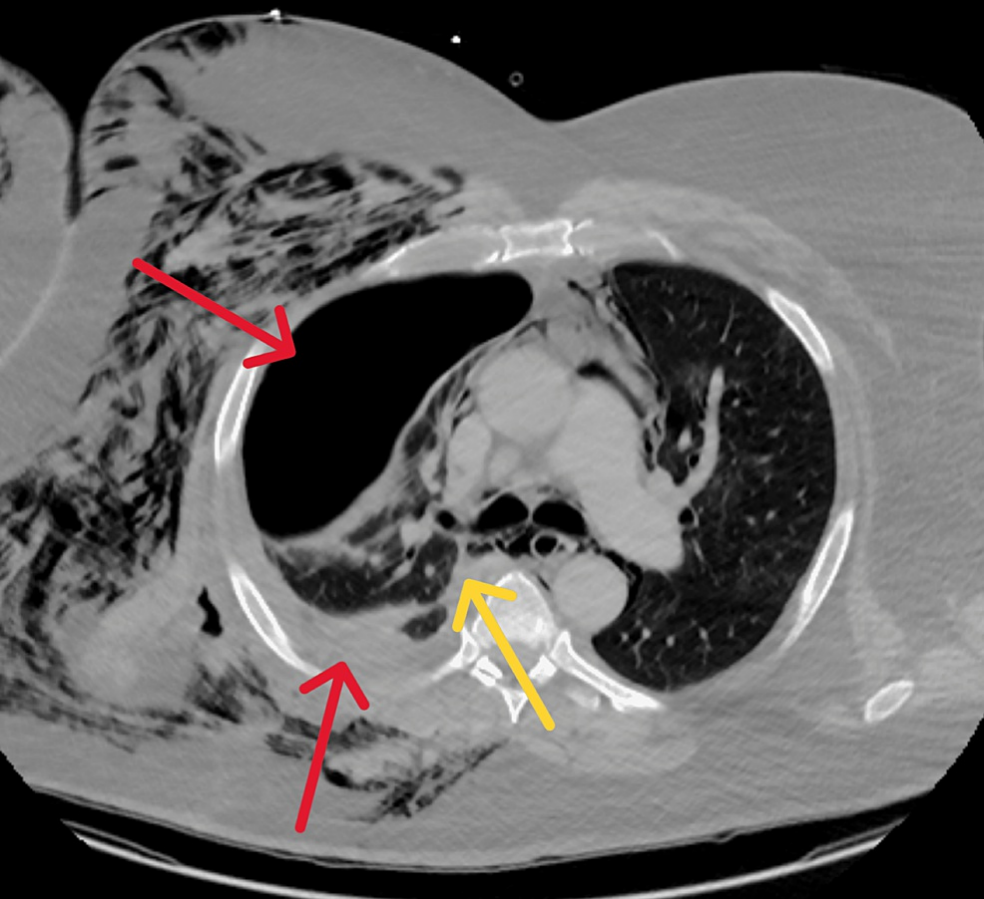
Computed tomography (CT) chest with contrast The red arrow shows a large right-sided hydropneumothorax comprising approximately 50% of the right hemithorax volume. The yellow arrow shows the development of pneumomediastinum.

Three days later, the patient was readmitted for worsening right-sided pneumothorax with CT with contrast chest showing large right-sided hydropneumothorax comprising approximately 50% of right hemithorax volume with a significant amount of subcutaneous emphysema extending into the neck, right upper extremity, and throughout the right chest. The development of pneumomediastinum was also noted (Figure 2).

**Figure 3:**
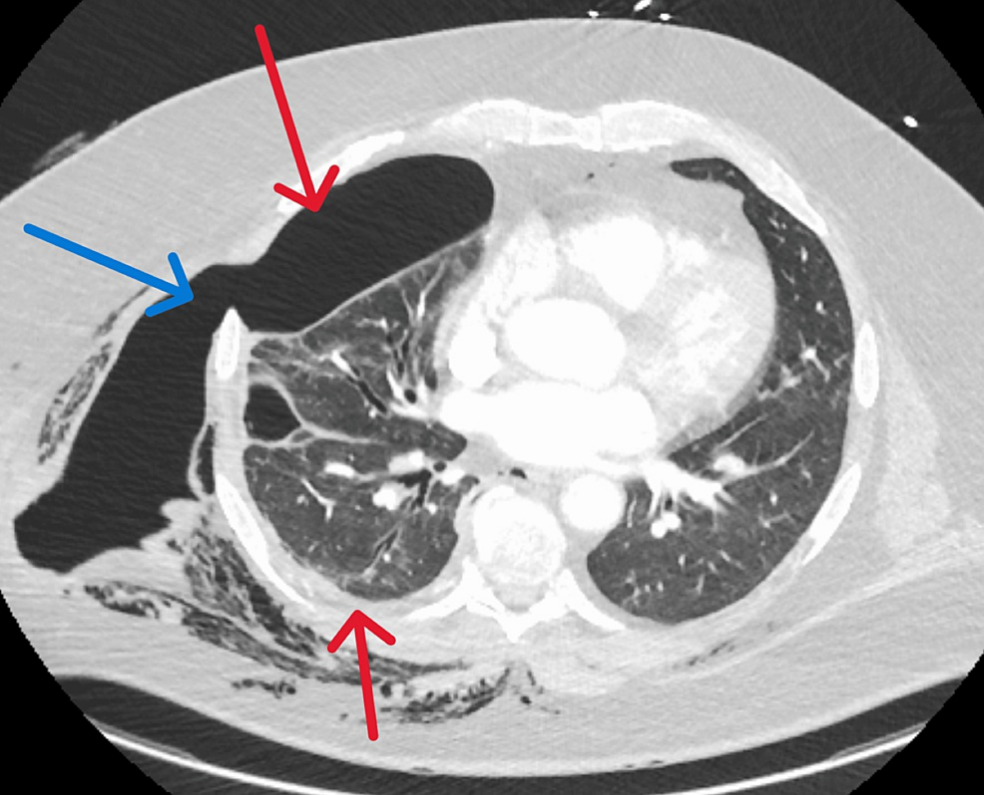
Computed tomography angiogram (CTA) chest The red arrows show the right hydropneumothorax. The blue arrow shows intercostal herniation of the pleura between the right fifth and sixth ribs out into the right lateral chest wall.

During this admission, cardiothoracic surgery placed the chest tube and left it in place for an extended duration of eight days with supportive care. The patient had persistent leukocytosis with no source of infection identifiable at this time and it was thought to be reactive as all the blood cultures had been negative since the first admission. On outpatient pulmonology appointment after five days of discharge, the patient appeared ill with worsening shortness of breath and increased work of breathing. He also stated that there was a "bulge in his chest" when he coughed. He underwent a CTA of the chest on arrival at the ED. This demonstrated an interval increase in the size of the right hydropneumothorax, with intercostal herniation of the pleura between the right fifth and sixth ribs out into the right lateral chest wall, along with persistent profound subcutaneous emphysema and pneumomediastinum (Figure 3).

**Figure 4:**
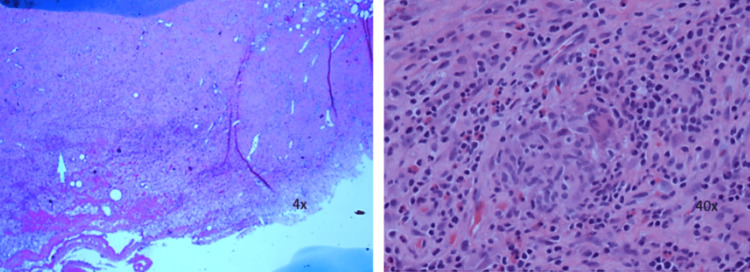
Surgical pathology Evidence of noncaseating granulomas on surgical pathology.

**Figure 5:**
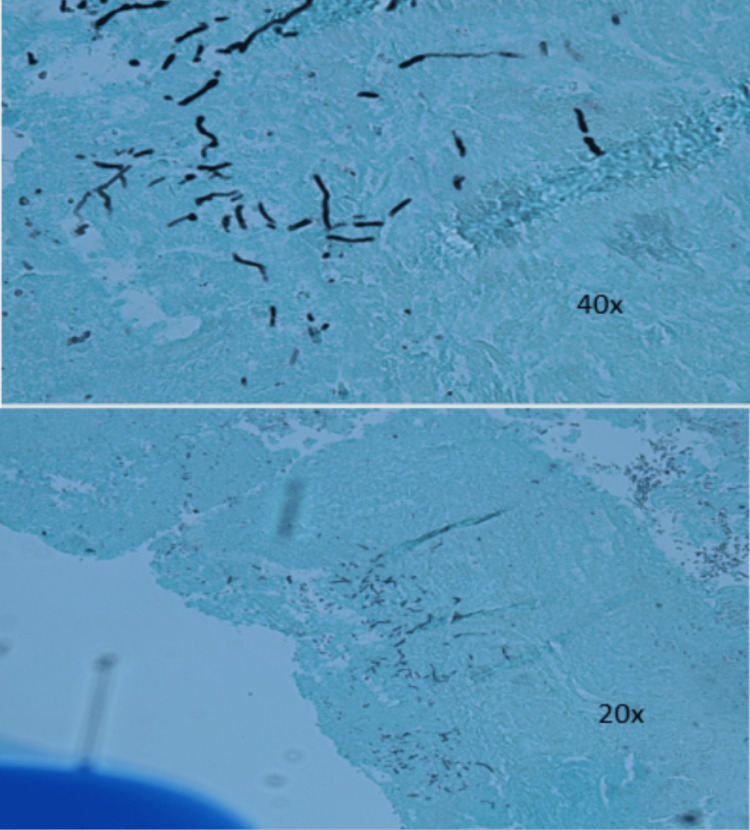
Gram stain Positive Gram stain showing fungal hyphae with septae and predominately 45-degree angle branching consistent with aspergillosis.

Surgical exploration and an attempt at the closure of his chest wall defect were planned by cardiothoracic surgery. On the reopening of previous right thoracotomy incisions, some sternal stitches were separated anteriorly with the rather large subcutaneous "hernia sac." The hernia sac was excised with electrocautery and multiple intercostal stitches were placed to reapproximate the chest wall. The surgical pathology report from the excised tissue of the right intercostal region showed pieces of benign fibro adipose tissue with fibrovascular proliferative changes, fat necrosis with acute, subacute, and chronic inflammation, and was negative for neoplasm. Small, noncaseating granulomas were present. Positive fungal hyphae were present showing septae and predominately 45-degree angle branching consistent with aspergillosis (Figures 4, 5).

**Figure 6:**
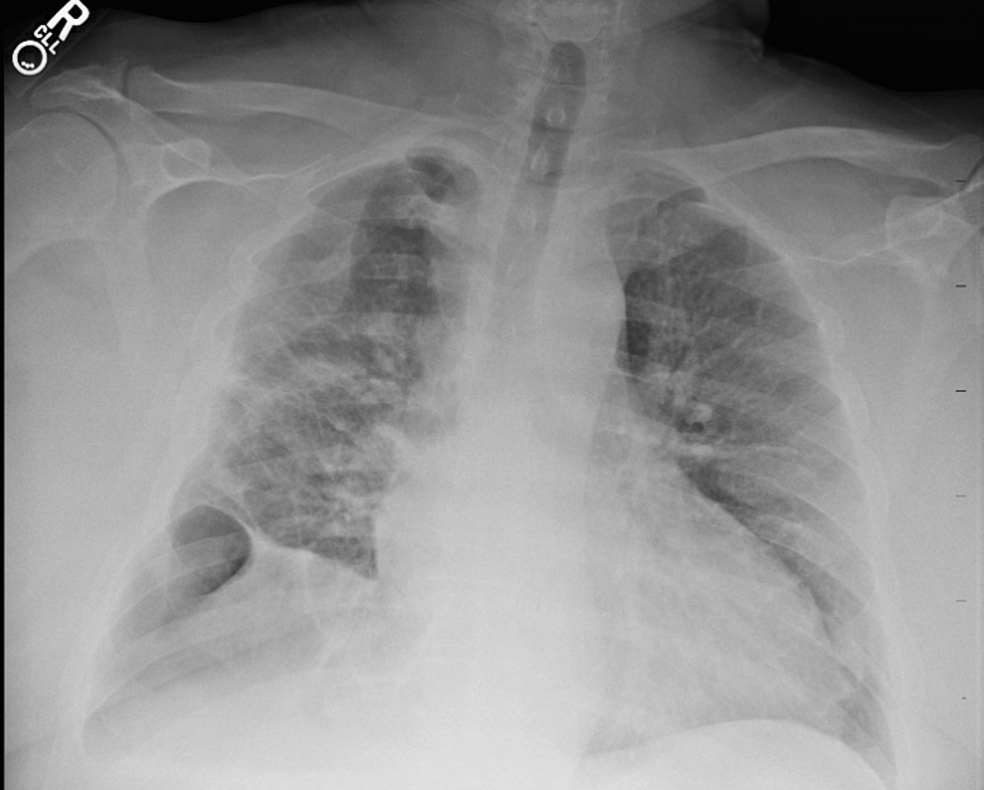
Postoperative chest X-ray at the outpatient clinic Chronic-appearing postoperative pleural parenchymal scarring laterally in the right hemithorax from the recent surgery. There is a trace residual volume of pleural fluid in the right lung base.

At this time infectious disease was consulted and the patient was started on voriconazole 200 milligrams per oral two times daily. Voriconazole levels were monitored every two weeks at the outpatient clinic and the patient was educated about the possibility of vision changes with the use of voriconazole. Serum Aspergillus antigen, serum Aspergillus antibody, Aspergillus niger antibody IgE, Fungitell 1-3 beta D glucan assay, and serum IgE level were all negative. Infectious disease monitored patient on voriconazole for six months. After initiating this medication, the patient's last chest X-ray showed stable findings of his chronic disease process with no new or worsening hydropneumothorax (Figure 6).

## Discussion

Historically, an established empyema and a bronchopleural or pleurocutaneous fistula were the two clinical settings most associated with pleural aspergillosis [4]. Pleuropulmonary aspergillosis is a rarely reported disease. Less than 1% of pleural effusions are caused by fungal infections [5]. It is difficult to pinpoint the exact time of our patient contracting aspergillosis in between invasive procedures as patients presenting with COVID-19 pneumonia and concomitant aspergillosis may not have typical findings such as nodules and cavities but will often have radiographic findings including ground-glass opacities, consolidations, and bronchiectasis. Predominant COVID-19 pneumonia causes ground-glass opacities, and early data suggest that cavitary lesions and nodules can be obscured due to these opacities [6,7]. Neutropenia is also a risk factor to be considered for invasive aspergillosis. CT halo sign is seen for a shorter period and is effective for diagnosis in the early disease process [6,8,9]. Other risk factors are other conditions causing profound immunocompromised state, malignancy, and history of organ transplantation [2]. In nonresponsive empyema, fungal cause also should be included in the differential diagnosis. Early diagnosis and treatment with appropriate antifungals and surgical intervention improve the outcome. Monitoring for the side effects of antifungal therapy is important. The management of pleuropulmonary aspergillosis in pregnancy can often be challenging and requires a dedicated multidisciplinary approach. A close outpatient follow-up either with the primary care provider or a pulmonologist or an infectious disease is required to navigate the treatment course as these patients require prolonged antifungal therapy. Monitoring patients for treatment response and toxicity leads to better outcomes and decreases disease burden as in our patient in this case. The choice of antifungal is broad-spectrum voriconazole either intravenous or oral preparation. It has better tolerability than traditional amphotericin B. Echinocandins are a class of antifungal agents that inhibit the synthesis of an essential component of the cell wall known as 1,3-β-D-glucan. Other agents from the same class of drugs include posaconazole and isavuconazole, which may be cautiously used due to the side effect profile. Physicians may use a combination therapy or a monotherapy depending on the patient's response. 

## Conclusions

Take-home points from this case would be to consider recurrent hydropneumothoraces as a rare manifestation of pleural aspergillosis infection but a strong suspicion of underlying dormant fungal infection should be on the differential diagnosis when recurrent episodes of pleural effusions are encountered. In this case, the patient's underlying comorbidities, poor baseline lung function with oxygen requirements, history of recent COVID-19 infection, and invasive procedures may have played a role in the pleural seeding of Aspergillus species. It is important for clinicians to recognize COVID-19 as a risk factor for pleuropulmonary infections, particularly in patients with comorbidities, structural defects like bronchopleural fistula, and chronic immunosuppressive therapy. Fortunately, our patient was not immunosuppressed and thus it is safe to assume that his condition did not progress into disseminated aspergillosis.
